# Transmigration route of *Campylobacter jejuni* across polarized intestinal epithelial cells: paracellular, transcellular or both?

**DOI:** 10.1186/1478-811X-11-72

**Published:** 2013-09-30

**Authors:** Steffen Backert, Manja Boehm, Silja Wessler, Nicole Tegtmeyer

**Affiliations:** 1 Department of Biology, Institute for Microbiology, Friedrich Alexander University Erlangen/Nuremberg, Staudtstr. 5, D-91058, Erlangen, Germany; 2 Department of Molecular Biology, Division of Microbiology, Paris-Lodron University of Salzburg, Billroth Str. 11, A-5020, Salzburg, Austria

**Keywords:** Adherens junctions, Cell polarity, E-cadherin, Fibronectin, HtrA, Integrins, Invasion, Molecular pathogenesis, Cellular invasion, Signaling, TER, Tight junctions, Transmigration, Virulence

## Abstract

Intact intercellular junctions and cellular matrix contacts are crucial structural components for the formation and maintenance of epithelial barrier functions in humans to control the commensal flora and protect against intruding microbes. *Campylobacter jejuni* is one of the most important zoonotic pathogens causing food-borne gastroenteritis and potentially more severe diseases such as reactive arthritis or Guillain–Barré syndrome. Crossing the intestinal epithelial barrier and host cell invasion by *C*. *jejuni* are considered to represent the primary reasons of gut tissue damage in humans and various animal model systems including monkeys, piglets, rabbits, hamsters and ferrets. *C*. *jejuni* is also able to invade underlying tissues such as the lamina propria, can enter the bloodstream, and possibly reach distinct organs such as spleen, liver or mesenteric lymph nodes. However, the molecular mechanisms as well as major bacterial and host cell factors involved in these activities are poorly understood. Various models exist by which the pathogen can trigger its own transmigration across polarized intestinal epithelial cells *in vitro*, the paracellular and/or transcellular mechanism. Recent studies suggest that bacterial factors such as flagellum, serine protease HtrA and lipooligosaccharide LOS may play an active role in bacterial transmigration. Here we review our knowledge on transmigration of *C*. *jejuni* as well as some other *Campylobacter* species, and discuss the pros and cons for the route(s) taken to travel across polarized epithelial cell monolayers. These studies provide fresh insights into the infection strategies employed by this important pathogen.

## Introduction

*Campylobacter jejuni* is a wide-spread Gram-negative bacterium living as commensal in the gut of most birds and domestic animals. However, *C*. *jejuni* is infectious for humans and consumption of contaminated food products is a major cause of human bacterial gastroenteritis, which may be responsible for as many as 400–500 million cases annually [1]. The clinical outcome of *C*. *jejuni* infection varies from mild, non-inflammatory, self-limiting diarrhoea to severe, inflammatory, bloody diarrhoea that can continue for few weeks [2-5]. In some cases, *C*. *jejuni* infections can be also associated with the development of reactive arthritis and peripheral neuropathies, known as Miller–Fisher and Guillain–Barrè syndromes [6,7]. Despite the significant health burden caused by *C*. *jejuni* infections, our present knowledge about the interplay between *C*. *jejuni* and its various hosts is still very limited. The availability of complete genome sequences from various *C*. *jejuni* isolates has started to improve our understanding in genetics, physiology, pathogenesis and immunity of *C*. *jejuni* infections in recent years. *C*. *jejuni* is the first bacterium reported to encode for both O- and N-linked glycosylation systems, a property that is likely influencing the host-pathogen crosstalk and disease outcome. In addition, a multitude of infection studies in various animal and *in vitro* cell model systems revealed the importance of *C*. *jejuni* motility and chemotaxis as critical features important for establishing successful infections [2,8-10]. In particular, the high motility (Mot^+^) permits *C*. *jejuni* to effectively move to its favored colonization niche at the inner mucus layer of the human intestine. Various *in vivo* and *in vitro* studies have shown that this pathogen encodes numerous virulence determinants involved in important disease-associated processes such as bacterial adhesion to, transmigration across, invasion into and intracellular survival within infected intestinal epithelial cells [11]. In the present article we review our current knowledge including various recent developments in *C*. *jejuni* research on how this bacterium can breach the gut epithelial barrier and transmigrate across polarised cell layers. In particular, we focus on the two major known routes that could be taken, the transcellular and paracellular ways of *C*. *jejuni* transmigration. Better molecular understanding of these pathways and the identification of involved bacterial and host factors is crucial for the future development of effective treatment regimes.

### The intestinal mucosa is a first barrier against microbial infections

**Figure 1:**
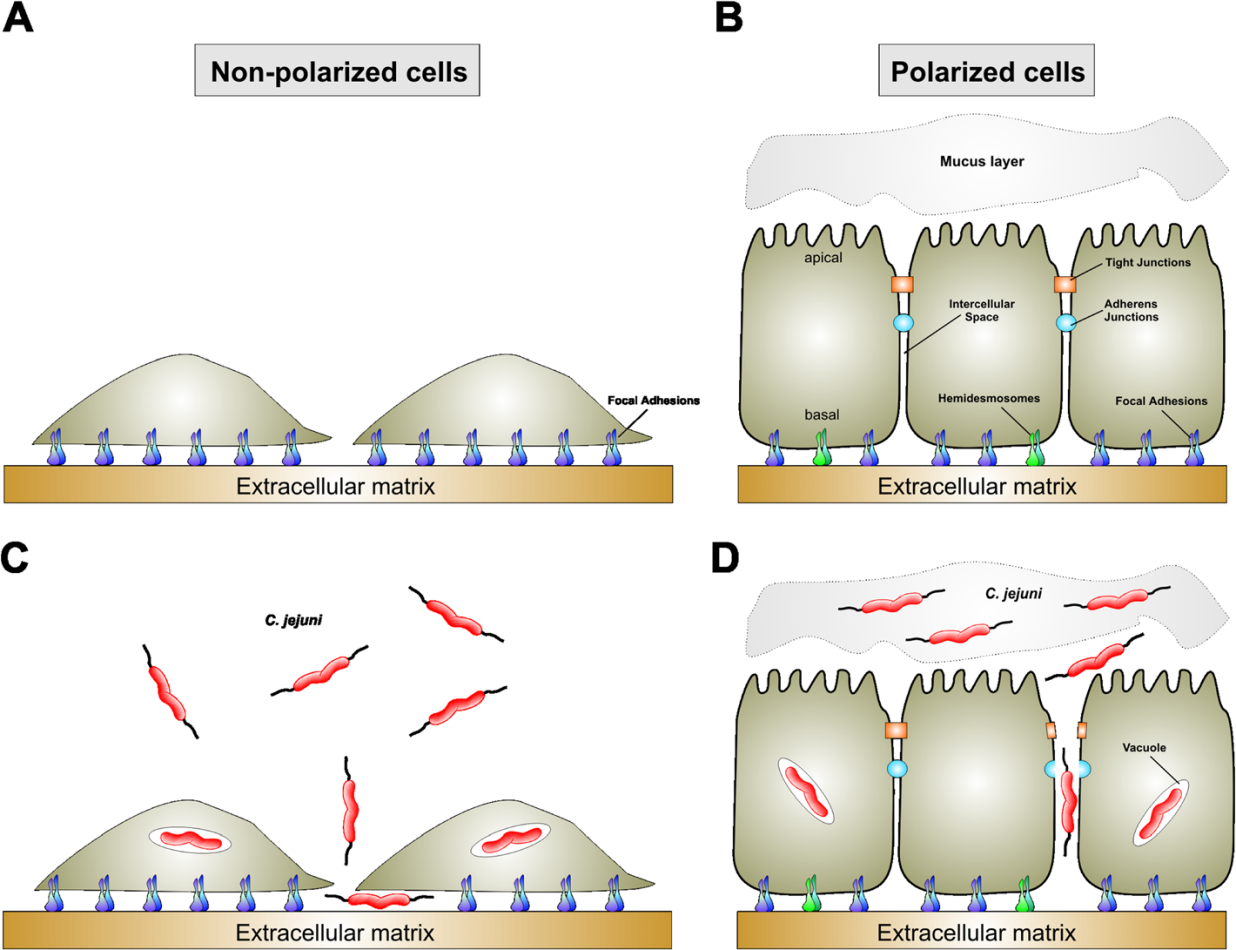
**A schematic presentation of non**-**polarised and polarized intestinal cell epithelial cells under non**-**infective conditions or during infection with *C***. ***jejuni*. (A)** Cultured non-polarised intestinal epithelial cells such as INT-407 do not express typical cell-to cell junctions. Thus, basolateral receptors such as focal adhesion structures are accessible and not protected by tight or adherens junctions. **(B)** Polarised intestinal epithelial cells such as mucin-producing HT29-MTX-E12 cells express the different types of intercellular junctions including the tight junctions (orange), adherens junctions (light blue), focal adhesions (dark blue) and hemidesmosomes (green) which exhibit specific localization in the lateral or basal membranes as indicated. GAP junctions and desmosomes are other examples which are not discussed in this review article. **(C**,**D)***C*. *jejuni* is able to infect both cell variants *in vitro*. This pathogen encodes numerous described pathogenicity-associated factors involved in important processes including bacterial adhesion to, transmigration across, invasion into and intracellular survival within intestinal epithelial cells. For more details see text.

**Figure 2:**
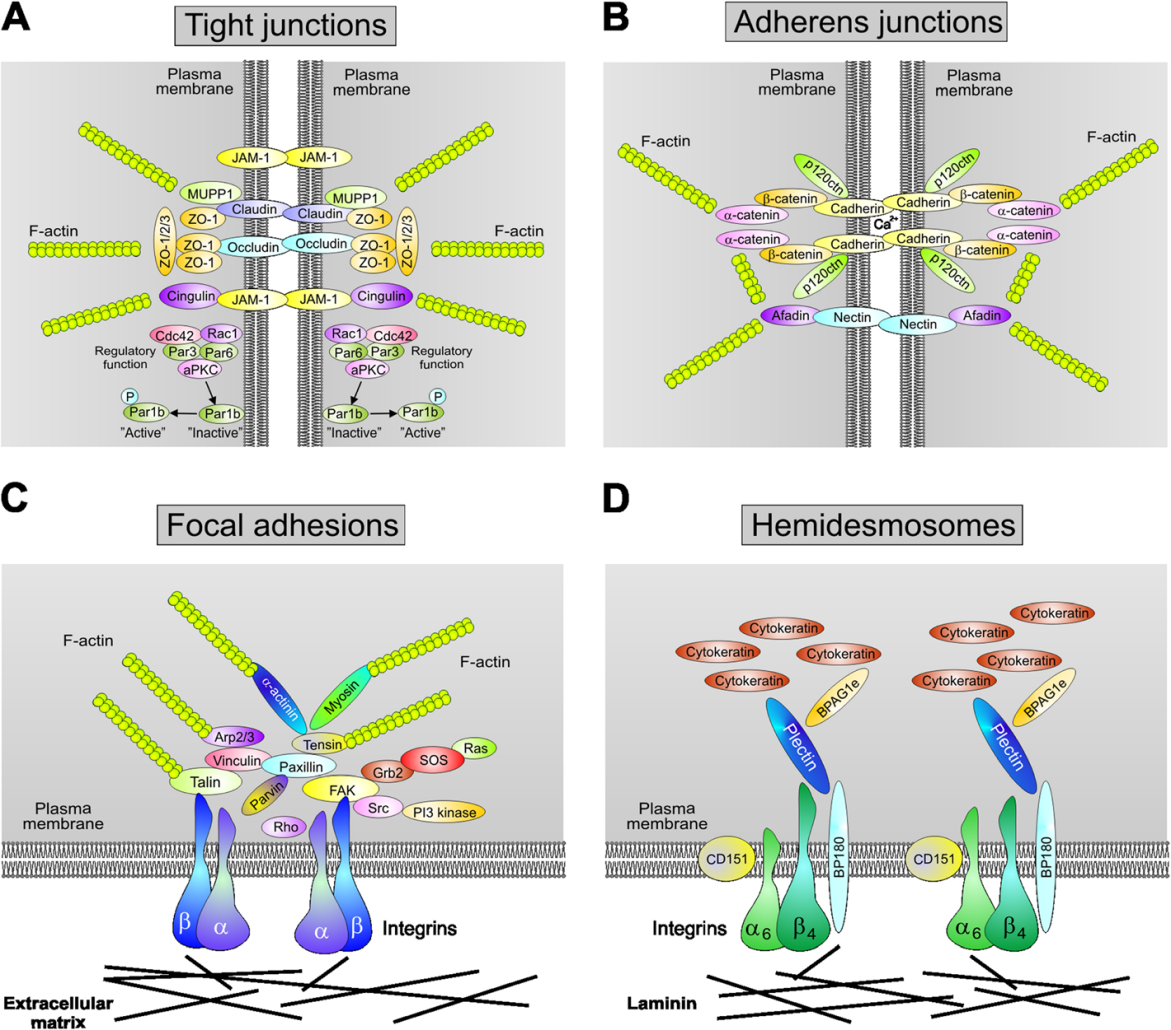
**Composition of major intercellular junctions in the polarized intestinal epithelium.** Schematic presentation of specific junctional complexes and associated signaling pathways. **(A)** Tight junctions (TJs) contain at least four major groups of transmembrane proteins: the JAMs, claudins, occludin and a number of cytoplasmic peripheral proteins. While the transmembrane proteins mediate cell-to-cell adhesion, the cytosolic TJ complex connects to different factors (e.g. ZO-1/-2/-3, MUPP1 or cingulin) that link the involved transmembrane proteins to the actin-cytoskeleton. The integrity of TJs is maintained by a regulatory complex including atypical PKC (aPKC), Rac1, Cdc42, Par6 and Par3. **(B)** The calcium-dependent integrity of adherens junctions (AJs) is stabilized by binding of E-cadherin to the intracellular catenins. The carboxy-terminal domain of E-cadherin binds to the cytoplasmic protein β-catenin. p120-catenin binds to the juxtamembrane part of E-cadherin and stabilizes the AJ complex. The E-cadherin-β-catenin structure is connected to the actin-cytoskeleton via binding to α-catenin and Eplin. When the E-cadherin complex is disrupted, β-catenin can translocate into the nucleus and activate Tcf/LEF transcription factors. **(C)** Focal adhesions (FAs) are structural complexes that link the extracellular matrix (ECM) to the intracellular actin-cytoskeleton. They contain various integrin heterodimers which are transmembrane receptors composed of α and β chains. The extracellular integrin tail directly binds to ECM proteins such as fibronectin, while the cytoplasmic domain is linked to the actin-cytoskeleton via a large number of indicated adapter/signaling proteins to transmit signaling. **(D)** Hemidesmosomes are also located at the basal side of epithelial cells where they link laminins to the intracellular intermediate filament network. Thus, hemidesmosomes provide stable adhesion of epithelial cell layers to the basement tissue. They consist of integrin α6β4, CD151 and BP180 which are transmembrane proteins, while plectin and BPAG1e are located in the cytoplasm. Plectin mediates linkage of hemidesmosomes to the cytokeratin network and not to F-actin filaments.

The intestinal mucosal epithelium in humans is an important cell layer that controls not only digestive, absorptive and secretory functions, but also forms the first barrier against pathogenic microbes [12]. The intact structure of healthy intestinal epithelial cells is maintained by the integrity of the apical-basal polarity, forming microvilli structures with a well-defined brush border, a highly organized actin-cytoskeleton and proper junctional complexes [13,14]. Importantly, well-established junctions are built up on the lateral cell-to-cell contacts including tight junctions (TJs) and E-cadherin-based adherens junctions (AJs) as well as basally located integrin-mediated cell-matrix contacts such as focal adhesions (FAs) and hemidesmosomes (HDs). While FAs are present both in cultured polarised and non-polarised cells, TJs, AJs, and HDs are only established in polarised and absent in non-polarised epithelial cells (Figure 1A,B). A model for the overall protein composition of these junction complexes is shown in Figure 2.

TJs are based on junction adhesion molecules (JAMs), claudins, occludin and other proteins, which represent important structural elements in establishing epithelial cell polarity. They are crucial for the tight sealing of the cellular sheets, thus controlling paracellular ion flux and therefore maintaining tissue homeostasis. The tight apposition of the membranes at TJs, which are localized at the apical end of the lateral membrane, also blocks lateral mobility of membrane proteins and lipids allowing the segregation of membrane components in an apical and basolateral compartment (Figures 1B and 2A). The AJs are positioned basal to TJs and form a network of membrane proteins and associated molecules, which are responsible for the mechanical adhesion between neighboring cells (Figures 1B and 2B). AJs assemble via homophilic, calcium-dependent interactions between the extracellular domains of E-cadherin on the surface of two adjacent epithelial cells. E-cadherin does not only act as an adhesive protein, but also has important functions as a regulator of cell proliferation. By modulating the availability of β-catenin, which binds to the intracellular domain of E-cadherin and helps to connect AJs with the actin cytoskeleton, E-cadherin-based AJs are involved in cell signaling and transcriptional regulation. Therefore, disturbed E-cadherin signaling is also associated with tumorigenesis [15]. The FAs comprise the third group of cell adhesion structures and consist of integrin heterodimers (composed of α and β chains), which are transmembrane receptors that link the extracellular matrix to intracellular FA proteins (Figures 1B and 2C). FAs modulate multiple signaling cascades to regulate cell attachment, proliferation, migration, differentiation and gene expression events. These processes are controlled by classical ‘outside in’ and ‘inside out’ signal transduction pathways [16,17]. The extracellular domain of a given integrin can directly bind to extracellular matrix proteins such as fibronectin, while the cytoplasmic tail is linked to the actin-cytoskeleton via a large number of adapter proteins, including vinculin, paxillin or talin, and signaling enzymes such as focal adhesion kinase (FAK) or Src kinase (Figure 2C). These protein complexes continually assemble and disassemble, and this turnover process must be differentially controlled at the leading edge versus the trailing edge of a migrating cell. In addition, HDs constitute adhesive protein complexes that mediate stable attachment of basal epithelial cells to the underlying tissues [18]. Similar to FAs, the organization of HDs relies on a complex network of protein-protein interactions, but in HDs integrin α6β4, laminin and plectin play essential roles (Figures 1B and 2D). Interestingly, many microbial pathogens including *C*. *jejuni* have adapted mechanisms during evolution to exploit TJs, AJs, FAs and/or HDs in infected cells in order to proliferate, survive and sometimes persist within the host [12,19-21].

### Detection of *C. jejuni* in the intestinal mucus, lamina propria, blood and other organs during infection *in vivo*

A major goal of current *C*. *jejuni* research is to define the exact role of bacterial adhesion, invasion and transmigration across enterocytes for the induction or absence of pathogenesis in different hosts. Several *in vivo* studies of human biopsies and infected animal models reported on observations of *C*. *jejuni* entering gut epithelial cells and underlying subepithelial tissues during infection (Table 1). For example, electron microscopic studies of biopsies from patients with campylobacteriosis have shown that *C*. *jejuni* can closely associate to the surface or within the intestinal epithelium, especially in Goblet cells, and was focally present in the lamina propria [22]. The majority of patients exhibited the histological picture of acute infectious colitis associated with massive infiltration of immune cells and marked distortion of crypt architecture. Penetration of *C*. *jejuni* into the intestinal tissue is also supported by the presence of blood and leukocytes in stool samples. Similar observations were obtained during *C*. *jejuni* infection experiments in monkeys [23], hamsters [24], piglets [25], rabbits [26] and ferrets [27]. In addition, live *C*. *jejuni* were recovered from other organs in infected animals such as the spleen [28-30], liver [27,29,30], mesenteric lymph nodes [29] and blood [26]. This suggests that *C*. *jejuni* exhibits the capability not only to adhere to and enter into enterocytes, but can also travel within the host, pass the intestinal epithelial barrier, enter the lamina propria and even access other organs of various infected hosts. Interestingly, a mixed set of results were obtained from infection experiments in chicken and mice. In multiple studies *C*. *jejuni* was regularly seen attached to or within colonic epithelial cells, lamina propria and other organs of chicken and mice [28-31]. However, *C*. *jejuni* infection resulted either in no obvious pathology or in less pathology as compared to humans or the above discussed other animal model systems (Table 1). In contrast, in some other reports *C*. *jejuni* was not seen attached to or inside intestinal epithelial cells, although high loads of bacteria were noted in the corresponding extracellular mucus layer [32,33]. These studies suggest that adhesion of *C*. *jejuni* to and invasion into intestinal epithelial tissues occurs *in vivo*, but may vary substantially in various chicken and mouse model systems, probably depending on the *C*. *jejuni* strain, variant and age of animals and other infection parameters (Table 1).

**Table 1:** **Selected reports on observations of *C***. ***jejuni *entering gut epithelial cells**, **underlying tissues or even other organs during infection *in vivo***^a^

**Host**	**Host sample and infection characteristics**	***C. jejuni *strains and characteristics**	**Applied methods**	**Disease symptoms/ macroscopic observations**	**Disease**-**associated molecular processes**	**References**
Human	Colonic biopsies (taken from 22 naturally infected patients), 3–30 days after onset of symptoms	Natural *Cj* strains	SEM, IHC, IFM	Acute infectious colitis with bloody diarrhea and *Cj*-positive stools; variation of IgA, IgM and IgG levels	Massive infiltration of immune cells; marked distortion of crypt architecture; invasion of *Cj* into colonic epithelial cells, Goblet cells and lamina propria	[22]
Hamster	Golden Syrian hamsters (age NP), 12 females, infection period: 12 days	*Cj* strain 4–82 (from human diarrheal stool)	TEM	Infection of ileum and cecum; diarrhea; intestinal and cecal abnormalities; 1 hamster died	Microvilli and cytoplasmic lesions; penetration of *Cj* into lamina propria, some intra-cellular; swollen ER; enlarged mitochondria	[24]
Piglets	Newborn piglets (2–4 weeks old), 10 animals, infection periods: 3–6 days	*Cj* strain M129 (campylobacteriosis patient)	TEM, LM, IHC	Bloody diarrhea; subacute, diffuse, mild to moderate, erosive colitis and typhlitis	Gross lesions of large intestine (not small intestine); cell damage with disrupted microvilli, *Cj* detected within cells and in lamina propria	[25]
Rabbits	New Zealand White rabbits (7–9 weeks old), 8 animals, infection period: 18 h	*Cj* strains L115, C119, O81 and P71(from inflammatory diarrhea)	IHC, LFA, GM1-ELISA	Gut tissue oedema, cell damage and submucosal bleedings	Massive infiltration of immune cells; high concentrations of enterotoxin, recovery of live *Cj* from blood	[26]
Monkeys	*Macaca mulatta* (3.5 month old); 2 infant monkeys, infection period: 17 days	*Cj* strain 78–37 (from human bloody diarrhea)	TEM	Colon damage and diarrhea	Intracellular and extracellular *Cj* in mucosa and basal lamina; exfoliated epithelial cells; some with apoptotic signs or dilated ER	[23]
Ferrets	*Mustela putoris furo* (5.5-6 weeks old ferrets); 15 females, infection period: 1, 2, 3, 6 and 9 days	*Cj* strain CG8421 (from human diarrheal stool); 81–176 (sequenced human isolate)	CFU-D, IHC, TEM	Acute infectious colitis with bloody diarrhea; *Cj* positive stools; variation of IgA-ASC, IgA, IgM and IgG levels	Massive colonization of small and large intestine; infiltration of immune cells; *Cj* within or between enterocytes; recovery of live *Cj* from liver	[27]
Mouse	BALB/c, C57BL/6 and DBA/2 mice (10 week old, both sexes); infection course: 24 days	Human *Cj* strains (from diarrheal stool)	CFU-D, IHC	None	*Cj* spreading and tissue invasion, recovery of live *Cj* from liver and spleen	[28]
Mouse	Myd88^−/−^ knockout mice (6–8 weeks old); infection period: 2, 4, 7, 9 and 14 days	81–176	CFU-D, ELISA, IB	Persistent *Cj* colonization of the intestine (but not in Myd88^+/+^ positive mice)	Impaired Erk activation and TNF-alpha/IL-6 cytokine production, recovery of live *Cj* from spleen, liver and mesenteric lymph nodes	[29]
Chicken	DeKalb X-L Leghorn chicks (1 day old), 170 animals, infection period: 14 days	A.J. and E.L. (human isolates); Ch-1 (chicken isolate)	CFU-D, SEM, IHC	Bloody diarrhea in 5 out of 16 1-day old chicks (start on day 2–5, recovered after 14 days); no symtoms in 3-day old chicks	*Cj* throughout the intestine; highest CFU in caecum and large intestine; both the upper and lower GI tract with inflammatory cells; *Cj* detected within cells and in lamina propria	[31]
Chicken	White Leghorn chicks (day of hatch); 41 animals, infection period: 14 days	RM1221	CFU-D, SA, CVM	None	Jejunal atrophy but no neutrophil infiltration or inflammation in the intestine; recovery of live *Cj* from liver and spleen	[30]

^a^*Abbreviations*: *ASC* (antibody-secreting cells); *CFU* (colony forming units); *CFU*-*D* (*CFU* determination); *Cj* (*Campylobacter jejuni*); *CVM* (crypt-vilus measurements); *ELISA* (enzyme linked immunosorbent assay); *ER* (endoplasmatic reticulum); Erk (extracellular regulated kinase); *GI* (gastrointestinal); *GM*1 (a specific ganglioside); *IB* (immunoblotting); *IFM* (immunofluorescence microscopy); *IHC* (immunohistochemistry); *IgA* (immunglobulin A etc.); *IL*-6 (interleukin-6); *LFA* (loop fluid analysis); *LM* (light miroscopy); Myd88 (myeloid differentiation factor 88); *NP* (not provided); *SA* (sucrase activity measurement); *SEM* (scanning electron microscopy); *TEM* (transmission electron microscopy); *TNF-alpha* (tumour necrosis factor alpha).

### What is the advantage for *C. jejuni* to cross the epithelial barrier and infect underlying tissues?

The degree by which a given pathogen can translocate through an epithelial cell barrier and the bacterium’s fate beyond the local environment differs substantially between different known microbes. For example, *Salmonella typhi* rapidly translocates across a polarized monolayer, causing cellular disruption leading to a complete loss of cell monolayer integrity. In contrast, the transcytosis of *Salmonella typhimurium* across polarized cells results in minimal damage to the epithelial monolayer [34,35]. Presumably these observations reflect disease manifestation *in vivo*, where infections with *S*. *typhi* are commonly septic in patients whereas infections with *S*. *typhimurium* are commonly restricted to the intestinal mucosa. By comparison to *Salmonella*, infections with *C*. *jejuni* are usually less acute. Advantages for *C*. *jejuni* reaching the underlying tissues and submucosa include that the bacteria are no longer subject to peristaltic forces in the intestine and they may gain pronounced access to certain nutrients such as iron. In addition, invasive *C*. *jejuni* can achieve contact with a set of basal host cell receptors such as fibronectin, which are normally not present at apical surfaces. Another advantage could be that the intracellular environment is better protected to antibiotics as compared to the gut lumen. Finally, by causing inflammatory diarrhea in the intestine, *C*. *jejuni* can improve its own spread to find a new host. This is in agreement with observations that stools from patients are diarrheal and remain *C*. *jejuni*-positive for several weeks [2-5].

### Use of non-polarised and polarised cells to study *C. jejuni* infection *in vitro*

Among others, the *C*. *jejuni* isolates 81–176, NCTC11168, F38011 and 81116 are the most commonly used strains in laboratories for infection studies *in vitro* (Table 2). Infection experiments of cultured cell lines with *C*. *jejuni* have shown that the bacteria can bind to (Bind^+^), invade into (Inv^+^) and survive inside a defined intracellular compartment (Surv^+^), called the *Campylobacter*-containing vacuole. These phenotypes, have been reported for both *C*. *jejuni* infection of non-polarised and polarised epithelial cells (Figure 1C,D). Studies of the translocation capabilities of *C*. *jejuni* strains across an intestinal epithelium *in vitro* require tight polarized cell monolayers. Typical chosen cell lines expressing TJs, AJs and FAs include Caco-2 [36-40], T84 [41-43], MDCK-I [43] or MKN-28 [44,45]. In addition, some of the polarised epithelial cell lines such as HT29-MTX-E12 have been shown to produce a mucus layer and thus maybe also very useful as they could better mimic the natural environment in the intestine [46,47]. It has been described that while *C*. *jejuni* can adhere to different cell lines with similar extend, the bacterial invasion and transmigration capacities can vary considerably between the different cell lines [43,48-50]. It was proposed that *C*. *jejuni* can enter cultured epithelial cell lines of human origin with higher efficacy as compared to non-human cells, suggesting that the pathogen is particularly specialised for disease-triggering infection of the human host [48].

**Table 2:** ***In vitro *studies of *C***. ***jejuni *translocation across polarized epithelial cell lines using transwell assays**^a^

**Applied cell model**	**Time of cell differentiation/growth**	**Confirmation of proper TJs before infection**	***C. jejuni *strains used**	**Negative controls used**	**Applied methods to investigate transmigration**	***C. jejuni *factors involved**	**Host factors involved**	**Proposed transmigration route**	**TER values during transmigration**	**References**
Caco-2	10-14 days	TER (300–500)	78–27, 81116, M129, F38011	*E*. *coli* DH5α	TWA, TER, PIS, SEM, TEM	NP	NP	Paracellular and transcellular	Unchanged (within 6 h)	[40]
Caco-2	NP	NP	37 clinical isolates	*E*. *coli* DH5α	TWA, CAA, GPA, CTA	NP	NP	Paracellular	NP	[36]
Caco-2	10-14 days	NP	81116	*E*. *coli* DH5α	TWA, TER, CAA, GPA, IB	FlaA/B	NP	NP	Unchanged (5 h)	[37]
Caco-2	10-14 days	TER (250)	6 clinical isolates	*E*. *coli* DH5α	TWA, TER, CPT	NP	NP	Paracellular and transcellular	Small drop of TER (from 250 to 200), but loss after 24 h	[49]
Caco-2	7 days	TER (430)	10 clinical isolates	NP	TWA, TER, CAA, GPA	NP	NP	Paracellular and transcellular	Changes were strain dependent (6 h)	[38]
T84	NP	NP	81–176, F38011	*E*. *coli* MRF	TWA, CAA, GPA	NP	NP	Paracellular	NP	[42]
Caco-2	7 days	TER (>1000)	81–176, NCTC11168	NP	TWA, SEM, TEM, GPA	NP	NP	Paracellular and transcellular	NP	[39]
HCA-7, T84	8-10 days	TER (400–550)	19 clinical isolates	NP	TWA, GPA, TEM, LDH, MF, IL-8, PGE2	NP	NP	Paracellular	Unchanged (12 h)	[50]
Caco-2	17 days	TER (1056)	R27456	*E*. *coli* DH5α	TWA, GPA, EPA, PIS	NP	NP	Transcellular	Unchanged (48 h)	[84]
T84, MDCK-I	NP	TER (400–500)	NCTC11168, 81–176, TGH9011	NP	TWA, PIS, GPA, EPA, TEM	FlgF	PI3-K	Transcellular	Unchanged (24 h), drop after 48 h	[43]
T84	NP	TER (values NP)	81–176, CHR213	*E*. *coli* HB101	TWA, PIS, IFM	FlaA/B	Cholesterol, caveolin	Transcellular	Unchanged (4 h)	[85]
MKN-28	14 days	TER (130–150)	81–176, NCTC11168	*E*. *coli* DH5α	TWA, TER, CA, ECA, IB	HtrA	E-cadherin	Paracellular	Unchanged (24 h)	[44,45]
Caco-2	19 days	TER (values NP)	GB11, GB19	NP	TWA, IFM, GPA	Cst-II	NP	Transcellular	NP	[59]

^a^*Abbreviations*: *CAA* (cell adhesion assay), *CA* (casein assay); *CPT* (cell permeability test using ^14^C-Inulin labelling); Cst-II (sialyltransferase); *CTA* (cytotoxic activity assay); *ECA* (E-cadherin cleavage assays), *EPA* (epithelial permeability assay); FlaA/B (flagellin genes A and B); FlgF (flagellar gene F); *GPA* (gentamicin protection assay); *MF* ([^3^H] mannitol flux); *IFM* (immunofluorescence microscopy); *HtrA* (high temperature resistant protein A, a serine protease); *IB* (immunoblotting ); *IL*-8 (IL-8 measurement); *LDH* (lactate dehydrogenase test); *NP* (not provided in the study); *PGE*2 (prostaglandin E2 measurement by *ELISA*); *PI*3-*K* (phosphoinositid-3-kinase); *PIS* (pharmacological inhibitor studies); *SEM* (scanning electron microscopy); *TEM* (transmission electron microscopy); *TER* (transepithlial electrical resistance measurement, values given in Ohms per cm^2^); *TJ* (tight junction); *TWA* (transwell assay).

### Bacterial and host factors with proposed roles in *C. jejuni* adhesion and invasion of intestinal cells

Although the exact molecular mechanisms triggering adhesion of *C*. *jejuni* to intestinal epithelial cells are still not fully understood, several studies have provided evidence in recent years that this is a multifactorial process requiring the concerted activity of various *C*. *jejuni* factors, which are under much debate [2,11,51]. Application of the gentamycin protection assay (GPA) and other approaches led to reports of more than 20 bacterial gene products potentially mediating the interaction of *C*. *jejuni* with various host cell lines. These factors comprise numerous genes of the flagellar apparatus [52-56], *pseA* modifying flagellin with the acetamidino form of pseudaminic acid [57], lipooligosaccharide (LOS) biosynthesis gene *galE*[58], sialyltransferase *cst*-*II* gene [59], N-glycosylation genes *pglB* and *pglE*[60], capsule biosynthesis genes *kpsM* and *kpsE*[61,62], autotransporter CapA [63], Peb1-4 membrane proteins [64-67], serine protease HtrA [45,68,69] and the fibronectin-binding proteins CadF and FlpA [70-73]. In addition, the proposed lipoprotein Cj0497 [74], cytochrome c oxidoreductase SOR encoded by the genes cj0004c and cj0005c [75], CJIE1 prophage homologs [76], components of a type VI secretion system (T6SS) [77] and surface-exposed lipoprotein JlpA [78] have also been shown to influence *C*. *jejuni* host cell adhesion. However, it is not yet clear if all above factors contribute directly or indirectly to *C*. *jejuni*-mediated host cell binding. Another handicap is that some described adherence factors cannot be confirmed by other groups [11]. For example, JlpA is dispensable for the binding of *C*. *jejuni* to chicken-derived hepatocellular epithelial carcinoma cells [73]. However, mutagenesis of most of these adhesion-related factors also exhibited distinct defects in invasion of *C*. *jejuni* suggesting a possible positive correlation between bacterial adhesion and host entry events. One interesting exception is again the *jlpA* mutant. This mutant exhibits a reduced adherence phenotype by ~20% of wild-type level [78], but was not defective for host cell entry [73,79,80]. Taken together, while a considerable number of putative bacterial binding factors have been described for *C*. *jejuni*, there is a large gap in our knowledge on the corresponding host cell receptors.

### *C. jejuni* transmigration across polarised epithelial cells in transwell chambers: role of protein biosynthesis and temperature

Different well-known intestinal pathogens such as *Listeria*, *Shigella*, *Salmonella* or *Yersinia* have the capability to transmigrate across the gut epithelial barrier (Trans^+^ strains), gain access to deeper tissues, trigger cell damage and cause disease in humans. There are two general mechanisms how bacterial pathogens can overcome the epithelial barrier, described as the paracellular and the transcellular migration routes [34,35]. Pathogens utilising the paracellular mechanism break the TJ and AJ complexes and cross the epithelial barrier by passage between neighboring epithelial cells [12]. In contrast, some other pathogens specialised on the transcellular mechanism and invade epithelial or specialised M cells at the apical surface followed by intracellular trafficking and exit these cells at the basolateral membrane [81,82]. Studies on the translocation capabilities of *C*. *jejuni* across an intestinal epithelium layer *in vitro* have been performed with multiple strains and polarized cell lines grown in transwell chambers (Table 2). Migration of various Trans^+^*C*. *jejuni* strains from the apical compartment of transwells through polarized cells was confirmed by determination of colony forming units (CFU) obtained from the lower chamber, GPA and other functional assays. Application of chloramphenicol, a well-known inhibitor of bacterial protein biosynthesis, reduced the transmigration potential of *C*. *jejuni* significantly [40]. The failure of chloramphenicol to completely abolish translocation may indicate that some of the bacteria possess the factors necessary to facilitate penetration while others may have to synthesize such components *de novo*[40]. *C*. *jejuni* adherence, penetration and transmigration activities were also inhibited at lower temperatures when investigated at 20°C and 4°C as compared to 37°C [40]. These data suggest that adhesion, internalization and translocation of *C*. *jejuni* require active bacterial and host cell processes at optimal temperature. The current, common opinion is that *C*. *jejuni* can effectively transmigrate *in vivo* and *in vitro*, but the involved mechanisms (paracellular and/or transcellular) are controversial in the literature (Table 2) and will be discussed below.

### Role of the chosen polarised cell line and variability among wild-type *C. jejuni* strains

Most of the polarised cell lines applied to study *C*. *jejuni* transmigration were of human origin (Caco-2, HCA-7, T84 and MKN-28). The majority of utilised *C*. *jejuni* strains can markedly interact with these cells and actively transmigrate in large quantities within 1-6 h, while a negative control (non-pathogenic *Escherichia coli*) did not (Table 2). One of the most popular and well-investigated cell lines is Caco-2, and invasive wild-type *C*. *jejuni* strains such as 81–176, NCTC11168, F38011 and 81116 also revealed pronounced capabilities to transmigrate across polarised Caco-2 cells and represent typical Inv^+^/Trans^+^ isolates (Table 2). Based on these criteria, however, it is not possible to decide if the strains transmigrate either by the paracellular or transcellular pathway, respectively. Inv^+^/Trans^+^ strains could take either route while transmigrating non-invasive *C*. *jejuni* strains (Inv^-^/Trans^+^) would be limited to the paracellular pathway. Bras and Ketley [49] described 6 *C*. *jejuni* isolates exhibiting various phenotypes in the Caco-2 infection model including Inv^+^/Trans^+^_,_ Inv^+^/Trans^-^ and Inv^-^/Trans^+^ strains, respectively. This study demonstrates that at least some Inv^-^/Trans^+^*C*. *jejuni* strains are to abound, which should travel exclusively by the paracellular route. In addition, the study shows that Inv^+^/Trans^-^ strains exist. This means that invasive *C*. *jejuni* strains do not necessarily exit polarised epithelial cells at the basal membrane to complete the transmigration step, but obviously stay within the intracellular environment. In another study, transcytosis across polarized Caco-2 monolayers was seen in 18 of the 21 colitis-associated *C*. *jejuni* strains, compared with only 11 of the 23 isolates from non-inflammatory diarrhoea [36]. Interestingly, 6 strains from the latter group had the Inv^-^/Trans^+^ phenotype; again in such cases transcytosis can be unambiguously attributed to a paracellular passage [36]. Harvey and co-workers [38] compared 10 *C*. *jejuni* wild-type isolates and showed that they differ by at least 10-fold in invasiveness and transmigration across Caco-2 cells. *C*. *jejuni* transmigration did not quantitatively correlate with the intracellular invasiveness of these isolates and a similar repertoire of strains including Inv^+^/Trans^+^_,_ Inv^+^/Trans^-^ and Inv^-^/Trans^+^ isolates were found as described above. Taken together, these data suggest that different phenotypic wild-type *C*. *jejuni* isolates exist in nature and that bacterial transmigration capabilities may correlate with colitis disease outcome. However, more studies are certainly necessary to substantiate this hypothesis.

### Transepithelial electrical resistance during *C. jejuni* transmigration: changed or unchanged?

A well-established technique to confirm the presence of tight cell monolayers with proper TJs and to monitor changes in cell permeability during infection is measuring the transepithelial electrical resistance (TER) with electrodes [83]. For this purpose, polarized cell lines are commonly seeded and differentiated for up to 2–3 weeks in transwell chambers and TER values are followed over time and during the course of infection. However, in a few studies the cells were grown just for about 1 week or the growth period and TER values were not provided (Table 2). A common opinion is that bacterial transmigration by the paracellular route or toxic effects by the bacteria lead to disruption of TJ and AJ junctional complexes, consequently TER should drop, thus increasing cell monolayer permeability. Interestingly, while infection with *Listeria*, *Shigella*, *Neisseria* and *Salmonella* reduced TER substantially over time, infection with the *C*. *jejuni* Inv^+^/Trans^+^ strains 81–176, F38011 und NCTC11168 did not influence TER significantly [44,45]. Many other transmigration studies determined and followed TER during the course of *C*. *jejuni* infection [38,40,43,49,50,84,85]. In most of these reports, *C*. *jejuni* traversed the polarised cell monolayers without any apparent damage to the host cells. Commonly, within 4–8 h almost no difference was observed in the morphologic appearance of host cells and TER values of *C*. *jejuni*-infected versus non-infected monolayers [37,38,40,44,50,84,85]. Interestingly, both *C*. *jejuni* Inv^+^/Trans^+^ and Inv^-^/Trans^+^ strains traversed the Caco-2 cell monolayer without causing apparent cell damage as judged by TER [49]. This inability to measure TJ disruption led many researchers to hypothesize that *C*. *jejuni* cannot pass the epithelial barrier by the paracellular route, at least not at early times of infection [38,43,49,84,85]. In prolonged studies, some groups observed no TER changes even after 48 h of infection with *C*. *jejuni*[84], while others saw TER remaining unchanged until 24 h followed by a drop of TER either after 24 h [49] or 48 h [43]. The drop of TER *in vitro* indicates that opening of TJs and AJs by *C*. *jejuni* associated with massive cell monolayer disruption can occur at late times of infection. Immunofluorescence microscopy of polarized T84 monolayers infected for 24 h with *C*. *jejuni* revealed a redistribution of the TJ transmembrane protein occludin from an intercellular to an intracellular location associated with a change in phosphorylation [41]. Host cell degeneration is consistent with the tissue damage and inflammation found in many cases of campylobacteriosis *in vivo* (Table 1).

### Electron microscopy reveals *C. jejuni* within and between neighboring epithelial cells

**Figure 3:**
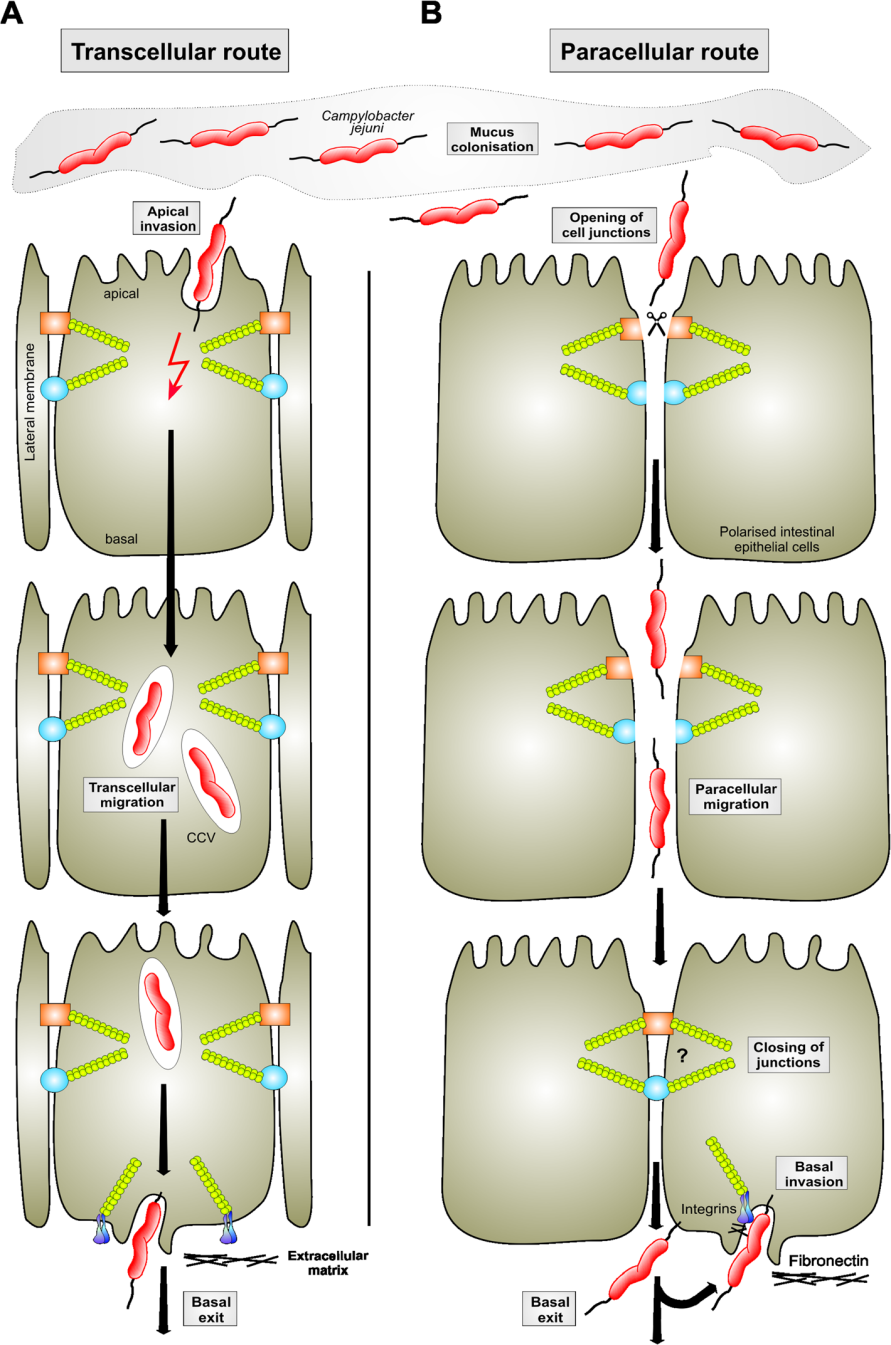
**Models for transepithelial migration across polarised epithelial cells by *C. jejuni*.** Simplified schematic diagram depicting cell junctions and two considered routes of bacterial travel across a polarized epithelium. The apical surface of the epithelial monolayer faces the external environment to the gut and forms the first barrier for *C*. *jejuni* invasion. Cell junctions important for the structural stability of a polarized epithelium include the tight junctions, adherens junctions, and matrix receptors as indicated. Various routes for *C*. *jejuni* transmigration have been proposed. **(A)** The transcellular route is characterized by pathogens crossing the epithelial barrier through entering the cells at the apical surface and exiting the cells at the basal membrane. **(B)** The paracellular route is taken by the bacteria entering the epithelium between two neighboring cells, thus crossing cells through the tight and adherens junctions. Opening of the cell-to-cell junctions maybe a temporal process and potentially close again after *C*. *jejuni* have passed. Basal exiting *C*. *jejuni* express the adhesin CadF which can bind to the fibronectin→integrin complex utilized for invasion from the bottom of epithelial cells.

Proper monolayers and junction formation in polarized cells when grown in transwells have been confirmed by scanning electron microscopy (SEM) or transmission electron microscopy (TEM), which also illustrated the presence of microvilli and well-defined brush borders at the apical cell surface [39,40]. In addition, immunofluorescence microscopy staining for JAM (a TJ marker) and E-cadherin (an AJ marker) was used to verify intact cell-to-cell junctions [44]. SEM and TEM studies were then applied to investigate the interaction of *C*. *jejuni* with polarized Caco-2 cells. The efficiency of *C*. *jejuni* invasion of Caco-2 cells was 2- to 3-fold less as compared to non-polarised INT-407 cells [39]. Interestingly, only 11-17% of differentiated Caco-2 cells were found to contain bound or internalized *C*. *jejuni*, and even smaller percentage of Caco-2 cells contained 5–20 internalized bacteria per cell after 2 h of infection [39]. Furthermore, SEM and TEM demonstrated that *C*. *jejuni* were present extracellularly between two neighboring Caco-2 cells as well as intracellularly [39,40]. Similar TEM observations have been obtained by some other researchers showing intracellular *C*. *jejuni*, which remain tightly surrounded by a host-derived membrane (the *Campylobacter* vacuole) after invasion into epithelial cells *in vitro* and *in vivo*[22,25,39,40,43,50,86]. It was therefore suggested that *C*. *jejuni* could translocate across polarized cell monolayers by passing through single cells (Figure 3A) and/or between two neighboring cells (Figure 3B).

### Bacterial and host factors involved in *C. jejuni* transmigration across polarised cells

The process of *C*. *jejuni* transmigration across polarised intestinal cells is not fully understood because only a handful putative bacterial and host factors have been reported yet. The application of pharmacological inhibitors has indicated that the activity of phosphoinositid-3-kinase is necessary for *C*. *jejuni* transcytosis [43]. The role of membrane lipid rafts was assessed by pharmacological depletion of cholesterol and caveolin co-localization using immunofluorescence microscopy [85]. In addition, it was shown that *C*. *jejuni* transmigration was enhanced by adding interferon-gamma, probably because of its TER-reducing capabilities during inflammation [87]. Many other studies have shown that inactivation of flagellar genes in *C*. *jejuni* resulted in a colonization-negative phenotype in various animal models [2,8,9]. Early studies using polarized Caco-2 cells and various flagellar and motility mutants indicated that either *C*. *jejuni* motility or the flagellin gene products or both appear to be essential for translocation across the polarized monolayer *in vitro*[37]. For example, *C*. *jejuni* mutants GRK5 and GRK7 (FlaA FlaB Mot^-^) and GRK17 (FlaA FlaB^+^ Mot^-^) were unable to cross the Caco-2 cellular barrier as compared to Mot^+^ control strains [37]. Another flagellin knockout (Δ*flaA*/*B*) and Δ*flgF* mutants with Mot^-^ phenotype were also diminished in passing polarised T84 or MKN-28 cells, respectively [43,44,85]. In addition, the wild-type strain NCTC12189, a *C*. *jejuni* variant that has reduced motility (Mot^−/+^) despite retaining intact flagella, was unable to colonise the intestinal tract of infant mice [88] and elicited no histological changes in the rabbit intestinal mucosa nor *C*. *jejuni*-positive blood culture [26]. It was therefore proposed that flagella and associated motility are the driving forces for colonization, invasion and transmigration properties of *C*. *jejuni*.

The flagellum does not only have a distinct function in bacterial motility and cell binding, but also acts as a type III secretion system (T3SS) for the delivery of Cia (Campylobacter invasion antigens) proteins into the extracellular space or into the host cell [89-93]. The first described Cia protein member is CiaB [89]. The CiaB protein was reported to be translocated into the cytoplasm of host cells, suggesting that it is a T3SS effector molecule facilitating invasion [89]. CiaB expression was also shown to be crucial for the secretion of at least eight other Cia proteins, ranging in size from 12.8 to 108 kDa, that were induced upon host cell contact or by the presence of calf serum [94]. However, the exact function of CiaB is not yet clear. Interestingly, the invasion-defective Δ*ciaB* mutant was able to transmigrate across polarised T84 cells like wild-type bacteria suggesting that apical cell invasion is not necessary for *C*. *jejuni* transmigration, thus favoring the paracellular route [42]. Further arguments for the paracellular route came from competition experiments with soluble fibronectin and observations that the Δ*cadF* mutant (deficient in fibronectin-binding and invasion) also transmigrated as effectively as wild-type *C*. *jejuni* strain F38011 [42]. This report is counteracted by another publication showing that a LOS-deficient Δ*cstII* mutant in *C*. *jejuni* strain GB11 exhibited a strong deficiency of invasion and transmigration as determined by GPA, immunofluorescence microscopy and transwell assays, thus favoring a transcellular route [59]. Unfortunately, in the latter two studies two different cell systems were used and TER was not followed over time (Table 2). Thus, differences in these observations are not yet clear, but could be explained by strain-specific properties.

### Role of serine protease HtrA and E-cadherin cleavage in *C. jejuni* transmigration

Recently, another factor was identified to be a novel virulence determinant in *C*. *jejuni*, the serine protease HtrA (high temperature resistant protein A) [69]. Deletion of the *htrA* gene in two strains resulted in strong deficiency of *C*. *jejuni* to travel across polarised MKN-28 cells [44,45]. This mutant was not affected with regard to motility and flagella production, suggesting that bacterial motility *per se* is not sufficient for *C*. *jejuni* transmigration [44], but *htrA*-mediated cell binding maybe involved [69]. Another important new discovery was that HtrA can be secreted into the cell culture supernatant by *C*. *jejuni*, although this class of proteases has well-known functions as chaperone and protein quality controllers in the periplasm of *E*. *coli*[44,45]. Secretion of HtrA by *C*. *jejuni* was enhanced during host cell contact or in the presence of calf serum, but was independent of the flagellar T3SS [95]. Infection studies and protease assays showed that HtrA cleaves the major AJ protein E-cadherin on epithelial cells and the recombinant protein *in vitro*[44,45]. Interestingly, E-cadherin cleavage has also been found for the HtrA proteins of other enteric pathogens including *Shigella*, *Helicobacter* and EPEC, but not for the urogenital pathogen *Neisseria*[45]. HtrA-mediated E-cadherin cleavage led to the disruption of AJs allowing *H*. *pylori* or *C*. *jejuni* to enter the intercellular space. In further studies, the HtrA ortholog in *H*. *pylori* has also been shown to cleave another protein, fibronectin, but *C*. *jejuni* HtrA has obviously lost this activity over fibronectin during evolution [44,96]. The molecular reason for this observation is unknown, however, it is in agreement with the concept that fibronectin is a major basolateral host factor necessary for *C*. *jejuni* binding to integrins and host cell invasion, at least during infection of non-polarised cells [42,71,72,97,98].

Deletion of *htrA* or substitution of *htrA* with a protease-deficient S197A point mutant in the bacteria resulted in severe defects for E-cadherin cleavage and *C*. *jejuni* transcytosis through MKN-28 cells [44,45]. Thus, cleavage of host junctional proteins like E-cadherin (and probably other yet unidentified host factors) by secreted HtrA could explain how *C*. *jejuni* may transmigrate intercellularly between neighboring cells using the paracellular route. Interestingly, in a time course of *C*. *jejuni* infection the total amount of cell-associated E-cadherin dropped to some extent, but did not lead to a complete cleavage, not even in 8h infections [44]. It was therefore proposed that cleavage of E-cadherin by HtrA could be strictly controlled, in a temporal and spatial manner, during infection. Such a localized action of the bacterial protease could possibly be achieved by restricting secretion of HtrA to the time point, when the bacteria attach to cell-cell adhesion sites [44]. As the host cell translation machinery continuously produces large amounts of E-cadherin, the host cells can quickly substitute cleaved proteins. This hypothesis could also explain why no significant reduction in TER was observed during infection with *C*. *jejuni* and suggests that these bacteria could somehow open and close the “door” between two neighboring cells [44]. Such a mechanism could be analogous to transendothelial migration of neutrophils, which transmigrate effectively from the bloodstream to the site of infection and do not cause any damage to the endothelial cells [99]. If our hypothesis turns out to be true, it may represent a clever novel infection strategy for transmigration of pathogens such as *C*. *jejuni* across polarised host epithelial cells.

### Translocation studies of other *Campylobacter* species

Studies on the epithelial transmigration of *Campylobacters* other than *C*. *jejuni* are rare in the literature, with only a few reports on *C*. *fetus*, *C*. *rectus* and *C*. *coli*. In a first study, the ability to translocate across epithelial barriers has been investigated during infection with *C*. *fetus*, a recognized pathogen of cattle, sheep and humans [100]. Using cultured Caco-2 cells, *C*. *fetus* was found to translocate efficiently within 24h without altering TER, similar to *C*. *jejuni* as discussed above. *C*. *fetus* was also observed to invade and subsequently egress from Caco-2 cells as shown in a modified GPA procedure and this occurred independently of *C*. *fetus* S layer expression [100]. SEM and TEM studies revealed the presence of *C*. *fetus* both at apical and basal surfaces as well as in intracellular locations, but not in the paracellular space. Pharmacological inhibitor studies demonstrated the requirement of a functional tubulin cytoskeleton, and together with the TEM data support a transcellular mechanism for *C*. *fetus* transmigration across Caco-2 monolayers [100]. Thus, the ability to invade and subsequently egress may contribute to establishing *C*. *fetus* infections in various hosts and can explain bacterial recovery from extraintestinal sites [100]. In a second report, *C*. *rectus*, a periodontal pathogen associated with human fetal exposure and adverse pregnancy outcomes including preterm delivery, was investigated [101]. Infection experiments in pregnant BALB/c mice have demonstrated that *C*. *rectus* can translocate from a distant site of infection to the placenta where it can induce fetal growth restriction and impairs placental development [101]. Infection with *C*. *rectus* was detected in 63% of placentas after two weeks and significantly decreased fetoplacental weight. In invasion assays, *C*. *rectus* was able to effectively invade human trophoblasts *in vitro* (but not trophoblasts of murine origin), and showed a trend for higher invasiveness as compared to *C*. *jejuni*. Interestingly, *C*. *rectus* infection significantly upregulated IL-6 and TNF-α levels in a dose-dependent manner in human trophoblasts, but not in murine cells, suggesting a correlation between invasion and cytokine activation [101]. It was proposed that the invasive trait of *C*. *rectus* in human trophoblasts may play a role in facilitating bacterial translocation and placental inflammation during early gestation [101]. A third report investigated *C*. *coli*, which is the dominant *Campylobacter* species commonly found in pigs [102]. An experimental trial was conducted to evaluate the colonisation and translocation ability of the porcine *C*. *coli* strain 5981 in weaned pigs over 28 days. Excretion of *C*. *coli* 5981 was seen for all piglets 7 days after inoculation and highest counts were detectable on day 10 [102]. Post-mortem, of luminal *C*. *coli* was observed for gut tissues of the small intestine and for the gut associated lymphatic tissues, such as jejunal mesenteric lymph nodes and tonsils as well as for spleen and gall bladder. In conclusion, this trial indicates that *C*. *coli* exhibit translocation and invasion capabilities in pigs making it a useful model system to study colonisation and pathogenicity of this pathogen [102].

### Concluding remarks

Different mechanisms are used by various enteropathogens to transmigrate across the host intestinal epithelium, including transcytosis through specialized M cells, phagocytosis by interepithelial leukocytes or transcytosis of enterocytes [35,81,82]. *C*. *jejuni* is a predominant zoonotic pathogen causing enterocolitis in humans worldwide. However, despite the high prevalence of *C*. *jejuni* induced disease and research progress made in recent years, our knowledge is still relatively limited as compared to other invasive pathogens such as *Salmonella*, *Listeria* or *Shigella*. A series of studies on human biopsies and animal infection experiments have demonstrated that *C*. *jejuni* is able to cross the intestinal epithelial barrier and enter underlying tissues, bloodstream and even other organs (Table 1). However, the mechanism of *C*. *jejuni* translocation is controverse and not well understood. There is only one report that M cells in the Peyer's patches may facilitate transport of *C*. *jejuni* from the intestine in rabbits [103]. From *in vitro* studies it seems clear that *C*. *jejuni* transmigration across polarised cultured cells requires *de novo* protein synthesis and depends on functional flagella. Unfortunately, there is no consensus on the transepithelial route followed by *C*. *jejuni*, both the transcellular and paracellular routes have been described (Table 2). If apical binding of *C*. *jejuni* to epithelial cells is a prerequisite for subsequent invasion and transcellular migration is also unclear. There is rapid increase in reports on putative bacterial adhesion factors – we have now a list of more than 20 bacterial factors with proposed role in binding and subsequent invasion [11]. In contrast, there is a large gap in our knowledge on corresponding host cell receptors. Thus, there is an urgent need for identifying and characterizing host receptors which can be attributed to certain bacterial factors. The only receptor pathway intensively studied and verified by various independent research groups is the CadF→fibronectin→integrin signaling cascade [42,71,72,97,98,104]. These studies have presented high resolution SEM pictures of various invading *C*. *jejuni* strains (showing details of the invagination process) in multiple non-polarised cell types [71,97,98], but corresponding qualitative and quantitative SEM data for a set of *C*. *jejuni* strains invading polarised cells from apical or basal membranes are currently not available. Alternative possibilities include the involvement of ganglioside-like LOS in apical invasion, thus favoring a transcellular route [59], but this model is in contrast to the paracellular model for HtrA-mediated opening of AJs and basal invasion as triggered by the CadF→fibronectin→integrin complex [44,45]. How *C*. *jejuni* can open the TJs after longer co-incubation times is yet unclear. Several studies exist that could support the apical invasion model, but can *C*. *jejuni* also enter host cells from basal surfaces? Basal engulfment and entry of *C*. *jejuni* into non-polarised Chang or polarised Caco-2 cells has been demonstrated by TEM and immunofluorescence microscopy, and this process has been called subvasion [79,105]. However, if paracellular transmigration is a prerequisite for subvasion in polarised cells is not yet clear. Furthermore, it is also unclear how the T3SS-dependent injection of certain Cia proteins fits in any of the above models. Thus, more studies are clearly required to unravel the sequence of events that allow *C*. *jejuni* strains to travel across polarised intestinal epithelial cells, either by a transcellular or paracellular pathway or a mix of both. It should be also considered that individual *C*. *jejuni* strains might switch from one to the other mode under specific culturing or infection conditions. Finally, besides the commonly applied transwell system, a vertical diffusion chamber model system has been recently described, which creates microaerobic conditions at the apical surface and aerobic conditions at the basolateral surface of cultured intestinal epithelial cells, thus producing an *in vitro* system that probably closely mimics *in vivo* conditions of the human intestine [106]. The use of this vertical diffusion chamber for studying the interactions of *C*. *jejuni* with intestinal epithelial cells demonstrated the importance of performing such experiments under conditions that converge to the *in vivo* situation and will allow novel insights into *C*. *jejuni* pathogenic mechanisms [106]. In addition, it should be noted that most of the cell lines used for *in vitro* studies are already transformed because they originate from cancer patients. A possible alternative option came from recent studies demonstrating that single Lgr5 (the receptor for the Wnt-agonistic R-spondins)-positive stem cells isolated from the intestine can grow in culture into complex epithelial organoid structures, called miniguts, which retain their original organ identity [107-109]. This important example of self-organization could be used for stem cell research and regenerative medicine, but also disease modeling of infections with enteric pathogens including *Campylobacter*. It therefore appears that transmigration of *C*. *jejuni* and that of many other *Campylobacter* species will continue to be a fascinating and rewarding research subject in the future.

## Competing interests

The authors declare that they have no competing interests.

## Authors’ contributions

SB performed the data collection and wrote the text. MB designed Table 1 and SW designed Table 2. NT was drawing the figures and wrote the figure legends. All authors discussed, read and approved the text including the final manuscript.
